# Using the Intrinsic Fluorescence of DNA to Characterize Aptamer Binding

**DOI:** 10.3390/molecules27227809

**Published:** 2022-11-12

**Authors:** Chang Lu, Anand Lopez, Jinkai Zheng, Juewen Liu

**Affiliations:** 1 Institute of Food Science and Technology, Chinese Academy of Agricultural Sciences, Beijing 100193, China; 2 Department of Chemistry, Waterloo Institute for Nanotechnology, University of Waterloo, Waterloo, ON N2L 3G1, Canada

**Keywords:** intrinsic fluorescence, DNA, aptamer, binding

## Abstract

The reliable, readily accessible and label-free measurement of aptamer binding remains a challenge in the field. Recent reports have shown large changes in the intrinsic fluorescence of DNA upon the formation of G-quadruplex and i-motif structures. In this work, we examined whether DNA intrinsic fluorescence can be used for studying aptamer binding. First, DNA hybridization resulted in a drop in the fluorescence, which was observed for A30/T30 and a 24-mer random DNA sequence. Next, a series of DNA aptamers were studied. Cortisol and Hg^2+^ induced fluorescence increases for their respective aptamers. For the cortisol aptamer, the length of the terminal stem needs to be short to produce a fluorescence change. However, caffeine and adenosine failed to produce a fluorescence change, regardless of the stem length. Overall, using the intrinsic fluorescence of DNA may be a reliable and accessible method to study a limited number of aptamers that can produce fluorescence changes.

## 1. Introduction

DNA aptamers have many advantages compared to antibodies, such as much lower cost, higher stability and ease of modification [1,2,3,4]. The majority of research has been focused on a few model aptamers, although hundreds of other aptamers have been published [5,6,7,8,9]. A main issue in the field is a lack of reliable yet readily accessible methods to measure aptamer binding [10,11,12]. The versatility of DNA-based assays has sometimes worked against it due to a lack of quality control [13]. While the majority of immunoassays require immobilization of antibodies or antigens [14], homogeneous assays are preferred for its simplicity and avoiding nonspecific binding to surfaces [15,16,17,18].

Fluorescence spectroscopy is probably the most commonly used method to characterize aptamer binding using either covalently attached fluorophores or using DNA staining dyes [19,20,21,22,23] and cationic polymers [24]. For example, aptamers can be terminally labeled with a fluorophore/quencher pair or a FRET pair to form an aptamer beacon [25]. Another reliable method is to design structure-switching aptamers, where a quencher-labeled complementary DNA is hybridized with a fluorophore-labeled aptamer [5,26,27]. These methods require expensive covalent modifications, making it difficult to study different aptamer sequences. Using DNA staining dyes is cost-effective, but this method is less reliable and sometimes has a small signal change [28].

Recently, intrinsic fluorescence of DNA has been reported [29,30,31]. Although quite weak, with a few µM of DNA, a decent fluorescence can be achieved, and this concentration is comparable to that used for CD spectroscopy and is much less compared to ITC [32]. In addition, DNA hybridization, G-quadruplex formation and i-motif formation have all been shown to induce DNA fluorescence change [33,34,35]. These reactions are accompanied with a large conformational change of DNA. Aptamer binding, on the other hand, might have less conformational change. Thus far, whether such fluorescence can be used to study aptamer binding remains to be explored. In this work, we performed systematic studies of a series of DNA oligonucleotides and aptamers. We found that the majority of the tested aptamers failed to induce a change in the intrinsic fluorescence, and only two examples appeared to be successful. 

## 2. Results

### 2.1. DNA Hybridization Drops Intrinsic Fluorescence

**Figure 1:**
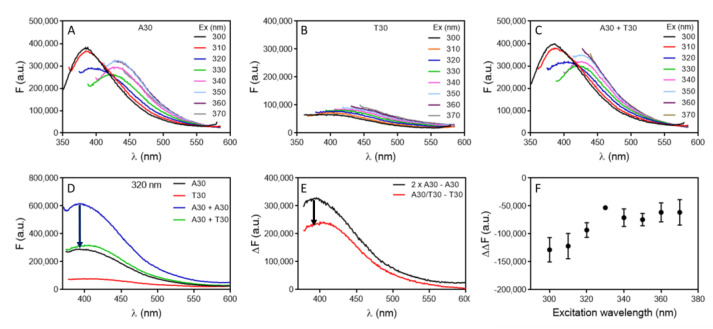
The fluorescence emission spectra of 5 μM (**A**) A30, (**B**) T30 and (**C**) annealed A30/T30 duplex excited at different wavelengths in buffer (10 mM PB, pH 7.0, 100 mM NaCl). (**D**) Comparison of fluorescence emission spectra of various DNA samples excited at 320 nm. (**E**) The fluorescence difference spectra: (2 × A30 − A30) and (A30/T30 − T30) with 320 nm excitation. (**F**) The difference of the difference spectra (the red line minus black line) in (**E**) at various excitation wavelengths.

In this study, we chose a few target molecules in order to obtain a comprehensive understanding of DNA intrinsic fluorescence for aptamer binding. Before testing aptamers, we first examined the DNA hybridization reaction, since complementary DNA can also be considered as a special aptamer target. We first studied the hybridization of A30 with T30. By varying the excitation wavelength of A30, we observed two emission peaks at 387 nm and 432 nm, respectively (Figure 1A). For T30, the fluorescence was weaker, and the emission peaks continuously varied with the excitation peak (Figure 1B). When an equal concentration of A30 and T30 were mixed, the fluorescence increased slightly compared to that of A30 alone (Figure 1C). However, part of the fluorescence increase was due to the extra T30 added. To test the effect of hybridization, we then measured the fluorescence difference of 10 µM A30 and 5 µM A30 (equation 1), and compared it with the fluorescence difference of 5 µM A30/T30 hybrid with 5 µM T30 (equation 2). If these two differences were equal, then DNA hybridization had no effect on the intrinsic fluorescence of DNA, and we only observed a simple sum of the two strands (Figure 1D). The reason to compare the difference instead of directly comparing A30 plus T30 with A30/T30 hybrids is to avoid potential interference from background signals in the spectra. When the fluorescence intensity is low, contributions from the background cannot be neglected.

(F_Background_ + 2 × F_A30_) – (F_background_ + F_A30_) = F_A30_ (1)

(F_Background_ + F_A30/T30_) – (F_Background_ + F_T30_) = F_A30/T30_ − F_T30_ (2)

Our results showed that the A30/T30 duplex DNA had a 27% lower fluorescence compared to the sum of the two strands (Figure 1E). We did the analysis for all the excitation wavelengths from 300 nm to 370 nm and the duplex fluorescence was lower at all the tested wavelengths (Figure 1F). Markovitsi and their coworkers studied the fluorescence of A20 and T20 DNA, and they found that with long wavelength UVA excitation (330 nm), the fluorescence of the A20/T20 duplex DNA was nearly 3-fold of their individual components [33]; however, we did not observe that. On the other hand, with UVC excitation (255 nm), the fluorescence yield dropped for the duplex, which was consistent with our observation, although we did not excite the sample at such a short wavelength to avoid the strong DNA absorbance at round 260 nm and hypochromicity associated with DNA hybridization. We did a control experiment with SYBR Green I (SGI) staining and confirmed formation of duplex DNA (Figure S1).

We then did the same experiment using a 24-mer random sequenced DNA named DNA1 and its complementary DNA (cDNA1), and dropped fluorescence was also observed upon hybridization (Figure S2). Therefore, we tend to believe that DNA hybridization would decrease the quantum yield of the intrinsic fluorescence of DNA when excited in the range of 300 nm to 370 nm. 

### 2.2. Cortisol Binding Enhances Aptamer Fluorescence

**Figure 2:**
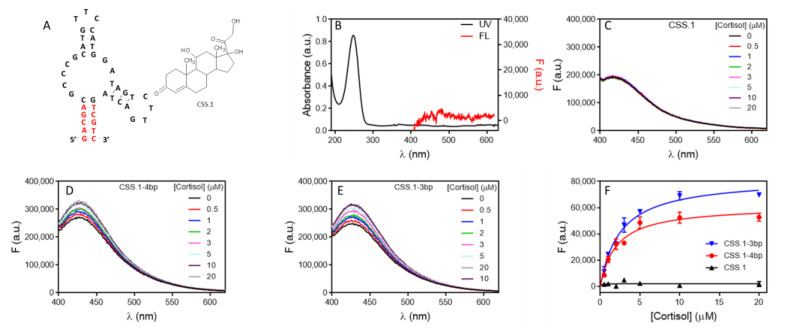
(**A**) The structure of the aptamer and cortisol. (**B**) UV-vis spectra of 50 uM cortisol and its fluorescence spectra when excited at 340 nm. The fluorescence of the (**C**) 5 bp (**D**) 4 bp and (**E**) 3 bp aptamers upon titration of cortisol. (**F**) The fitted binding curve of the three aptamers.

After understanding DNA hybridization, we then focused on aptamers. We first tested the cortisol binding aptamer for its high binding affinity (*K*_d_ ~100 nM) [27,36]. The secondary structure of the aptamer and the structure of cortisol are shown in Figure 2A. The UV-vis spectrum of cortisol is shown in Figure 2B and a peak at 247 nm was observed. In addition, it does not have intrinsic fluorescence (Figure 2B, red spectrum).

**Figure 3:**
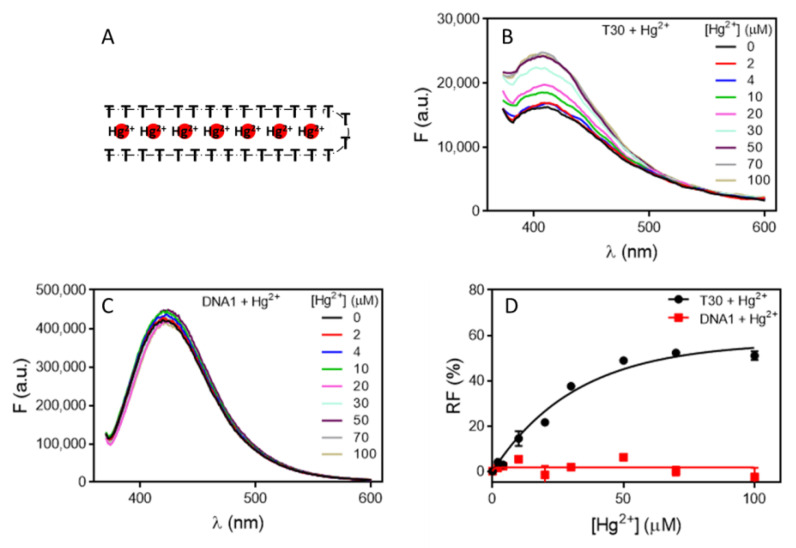
(**A**) A scheme showing Hg^2+^ binding to T30 DNA and each DNA was estimated to bind around 13 Hg^2+^ ions. The fluorescence spectra of (**B**) T30 and (**C**) DNA1 upon titration of Hg^2+^ with 320 nm excitation. (**D**) The fitted binding curve of the two DNAs.

We first tested the aptamer in Figure 2A. When excited at 340 nm, a fluorescence peak from 5 µM of the aptamer solution was observed at 416 nm. However, when titrated with cortisol, no fluorescence change was observed (Figure 2C). This aptamer has a 5 bp stem. We suspected that the aptamer was already folded, and cortisol binding only induced some minor local conformational changes, which was too small to affect the aptamer fluorescence. To test this hypothesis, we then tested the same aptamer but with the stem shortened. Interestingly, we observed cortisol-dependent fluorescence enhancement in both the 4 bp (Figure 2D) and 3 bp aptamers (Figure 2E). We reason that the shorter aptamers were initially in an open conformation, which was closed upon cortisol binding. It is interesting that the fluorescence enhanced in this case. Since we expected DNA duplex formation to decrease fluorescence, the increase was likely from the formation of non-canonical base interactions. For example, we observed 9-fold fluorescence increased when K^+^ was added to a G-quadruplex forming DNA [35].

The data were fitted and showed a similar *K*_d_ of around 2 µM (Figure 2F). Since the aptamer concentration was high (5 µM) compared to the expected *K*_d_ of 100 nM, the system is in the titration region and the *K*_d_ is roughly half of the aptamer concentration [37]. Therefore, for high affinity aptamers, it might not be possible to obtain an accurate *K*_d_. Nevertheless, it can be used to confirm aptamer binding, and to study the effect of other conditions, such as mutation. We also tested a few other DNA sequences and when cortisol was added, no fluorescence change was observed (Figure S3).

### 2.3. Hg^2+^ Binding to Poly-T DNA Enhances Fluorescence

We then tested Hg^2+^ binding using a polythymine DNA, T30 [38]. Hg^2+^ binding to T30 was verified by the SGI staining (Figure S4) [39]. Since T30 is not a sequence derived from an aptamer selection, it is technically not an aptamer but can still serve as an interesting model system. Hg^2+^ can specifically bind between two thymine bases forming a T–Hg^2+^–T base pair and fold the DNA into a hairpin structure (Figure 3A). This large conformational change of DNA may cause a fluorescence change. To test this hypothesis, we measure the fluorescence emission spectrum of T30 with 320 nm excitation, and the DNA showed an emission peak at 409 nm. The fluorescence increased when Hg^2+^ was titrated (Figure 3B), suggesting the T–Hg^2+^–T binding-directed the fluorescence change. This is quite striking since Hg^2+^ is known for its fluorescence quenching property. As a control, we also tested a 24-mer random sequenced DNA, which was not expected to bind to Hg^2+^. In this case, no fluorescence change was observed upon titrating Hg^2+^ (Figure 3C). Next, the relative fluorescence change of the two DNAs was calculated, and the intrinsic fluorescence of T30 increased up to 50% (Figure 3D). The fitted *K*_d_ for Hg^2+^ binding was 38 µM. Again, this is not the true *K*_d_ due to the high concentration of DNA used. Since each T30 DNA can bind around 13 Hg^2+^ ions (assuming a 4-nucleotide loop), half of that for 5 µM DNA is 32 µM, which is close to the observed *K*_d_.

### 2.4. Adenosine Binding Aptamer Fails to Show Aptamer Fluorescence Change

**Figure 4:**
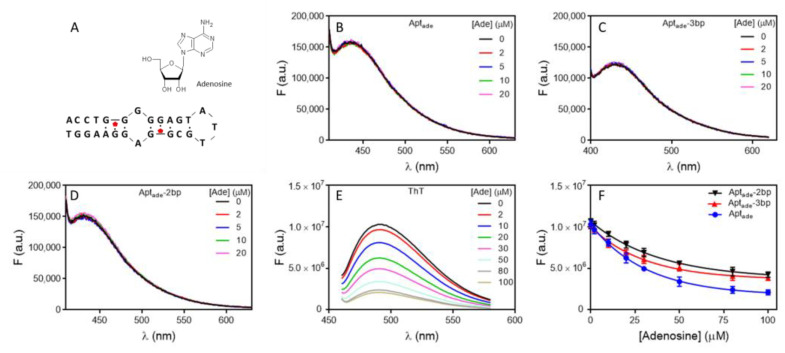
(**A**) The structures of the aptamer and adenosine. The fluorescence spectra of the (**B**) 4 bp (**C**) 3 bp and (**D**) 2bp adenosine binding aptamer upon titration of adenosine with 350 nm excitation. (**E**) The fluorescence emission spectra of ThT staining 100 nM of the adenosine aptamer after adding different concentration of adenosine in buffer (10 mM PB, 100 mM NaCl, 10 mM MgCl_2_ pH 7) with 450 nm excitation. (**F**) The fitted binding curves based on ThT staining of the three aptamers.

Next, the adenosine aptamer was studied, which is a model aptamer with a *K*_d_ around 7 µM [40]. The structures of adenosine and its aptamer are shown in Figure 4A. Adenosine has a UV absorption peak at 260 nm (Figure S5), and weak fluorescence when excited at various wavelength (Figure S6). We titrated adenosine to the aptamer at different excitation wavelengths (Figure S7), and similar trends were observed at all these wavelengths. To avoid the interference from adenosine absorption, we chose 350 nm as the excitation wavelength. Within 20 µM adenosine, no fluorescence change was observed for the aptamer (Figure 4B). We then shortened the aptamer to contain three or two base pairs in the stem. Surprisingly, we still did not observe any fluorescence change upon adding adenosine (Figure 4C,D). Next, we measured ThT fluorescence spectroscopy to verify the binding of adenosine and aptamer [41]. This aptamer is rich in guanine and can increase the fluorescence of associated ThT. Upon binding to adenosine, some ThT may be displaced from the aptamer/adenosine complex, leading to decreased fluorescence. Indeed, a large fluorescence drop was observed upon the addition of adenosine to the three aptamers (Figure 4E,F and Figure S8), confirming that the three aptamers can bind adenosine.

Given the structure of this aptamer, one would expect a large conformational change upon target binding, especially for the shortened aptamers. The fact that no fluorescence change was observed could be related to the canceling of the fluorescence enhancement and dropping factors.

### 2.5. Caffeine Binding Aptamer Fails to Show Aptamer Fluorescence Change

**Figure 5:**
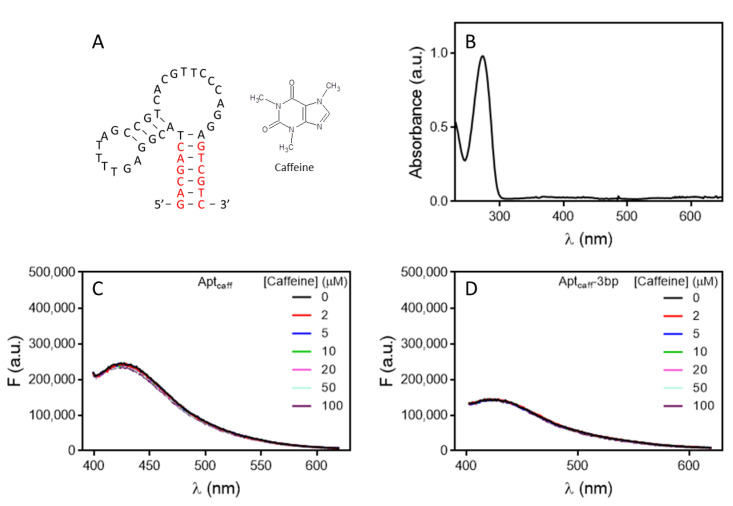
(**A**) The structures of the caffeine aptamer and caffeine. (**B**) UV-vis spectrum of 100 µM caffeine. The fluorescence spectra of the caffeine aptamer with (**C**) 6 bp and (**D**) 3 bp in the stem region upon titration of caffeine.

We recently reported an aptamer for caffeine [6]. Its structure is shown in Figure 5A. Caffeine has strong absorption in the UV region with a peak at 273 nm (Figure 5B), and it also has fluorescence when excited at 300 nm or 310 nm (Figure S9). The interference from the intrinsic fluorescence of caffeine was minimal when excited at 340 nm or longer (Figure S10). Therefore, we chose to excite the aptamer at 340 nm. When we titrated caffeine to the aptamer; however, no change in fluorescence was observed (Figure 5C), and when we truncated the stem down from even to just one base pair, still no change was observed (Figure 5D, Figure S11).

## 3. Discussion

**Figure 6:**
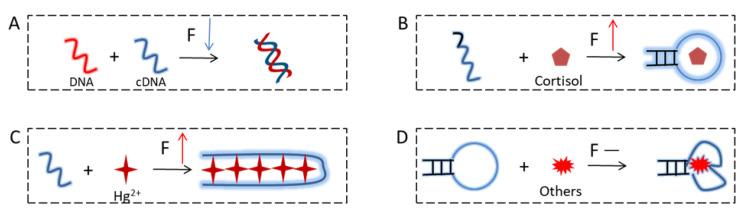
Schemes showing changes in DNA intrinsic fluorescence upon (**A**) DNA hybridization, and target binding to the (**B**) cortisol aptamer, (**C**) T30, and (**D**) the adenosine and caffeine aptamers that did not show a fluorescence change.

In this work, we examined the change in the intrinsic fluorescence of DNA upon hybridization and aptamers upon target binding. In three cases, we observed fluorescence change, while in the other two, we did not. We summarized our results in Figure 6. DNA hybridization decreased the fluorescence intensity (Figure 6A). The cortisol aptamer (Figure 6B) and Hg^2+^ aptamer (Figure 6C) showed binding induced fluorescence enhancement. However, adenosine and caffeine did not produce measurable fluorescence changes (Figure 6D). For typical small molecule binding aptamers, cortisol is the only example showing a fluorescence change, and the amount of fluorescence change was quite small. Even for the cortisol aptamer, when a stable aptamer with a long stem was used, no fluorescence was observed. Therefore, it is quite hard to predict the intrinsic fluorescence change of an aptamer upon binding to a small molecular target.

The question we intended to address in this work was whether the intrinsic fluorescence of aptamers could be used to study aptamer binding. Based on the above studies, the answer is likely to be no, especially for newly selected aptamers. A lack of target-dependent fluorescence change does not rule out aptamer binding. All the experiments here were performed using DNA and DNA aptamers. Since the fluorescence is related to DNA bases, we expect that RNA should have similar behavior.

For aptamers that show a fluorescence change, and if the fluorescence change is sufficiently large, this can be a cost-effective way to characterize aptamer binding and can provide useful information about binding kinetics, and buffer and salt requirement of binding. However, if the *K*_d_ is smaller than the DNA concentration, this method cannot be used to measure *K*_d_.

If forming Watson-Crick base pair drops DNA’s intrinsic fluorescence, then the increase we observed in the cortisol aptamer and T30/Hg^2+^ system was not due to the increase of the Watson-Crick base pair content. The reasons could be non-canonical base pairs and forming a more hydrophobic binding environment. This fluorescence increase in the cortisol case was quite small. For example, a large increase was seen when K^+^ was added to G-rich DNAs (up to 9-fold), while a few fold increase was observed when pH was dropped for an i-motif forming DNA [29].

It is also important to pay attention to the fluorescence of target molecules. Although most of the molecules are not considered to be fluorescent (adenosine, caffeine), they have detectable emissions at around 10 µM or higher if excited at the right wavelength (Figures S6 and S9). Thus, it is important to choose an excitation wavelength to avoid such interference.

## 4. Materials and Methods

### 4.1. DNA Hybridization Drops Intrinsic Fluorescence

All of the DNA samples used in this work were purchased from Integrated DNA Technologies (Coralville, IA, USA) and their sequences are listed in Table S1. Mercury acetate (Hg(Ac)_2_), magnesium chloride (MgCl_2_), fluorescein sodium salt, cortisol, adenosine, caffeine, and thioflavin T (ThT) were from Sigma-Aldrich. Sodium chloride (NaCl), sodium nitrate (NaNO_3_), sodium phosphate monobasic monohydrate, and sodium phosphate dibasic heptahydrate were obtained from Mandel Scientific (Guelph, ON, Canada). SYBR Green I (SGI) was purchased from Lonza (Rockland, ME, USA). Milli-Q water was used to prepare buffers and solutions.

### 4.2. Fluorescence Spectroscopy

All of the fluorescence spectra were recorded on a Horiba Fluoromax-4 spectrofluorometer (HORIBA Scientific, Edison, USA). The DNA was excited at 300–370 nm and its emission was recorded from 350 to 620 nm. 500 μL of DNA in buffer (100 mM NaCl, 10 mM MgCl_2_, 1 mM PB pH 7) was put in a 1 cm × 1 cm quartz fluorescence cuvette, and then different concentrations of targets were added for fluorescence measurement. The measurements in this work were carried out in triplicate and the standard deviations were plotted as the error bars. All the experiments were performed at room temperature (~22 °C) unless otherwise indicated.

## 5. Conclusions

This work examined the change of the intrinsic fluorescence of DNA and aptamers upon hybridization and target binding. In contrast with the large fluorescence changes observed for the formation of G-quadruplex structures and i-motifs in previous work, we observed very small changes and even no change for aptamer binding. Our study indicated a slight fluorescence drop upon DNA hybridization. There is an increase in the fluorescence of the cortisol aptamer and T30/Hg^2+^ systems, which were attributable to the formation of non-canonical base pairs. However, we did not observe fluorescence change for caffeine or adenosine aptamers, even if we truncated the stems to afford larger conformational changes. Given the vast number of aptamers published, it is impossible to test them all in one paper. This paper has shown a few different types of behaviors and careful controls are needed to understand whether this method can be used for new aptamers. For systems that have a reliable fluorescence change, this method can be an effective way to study aptamer binding. Understanding the reason for the (lack of) fluorescence change upon target binding could be a topic for future research.

## Data Availability

The data presented in this study are available on request from the corresponding author.

## Supplementary Materials

The following supporting information can be downloaded at: https://www.mdpi.com/article/10.3390/molecules27227809/s1, Table S1: The DNA Sequences Used in This Work; Figure S1: The fluorescence emission spectra of ssDNA or dsDNA stained by SGI; Figure S2: The fluorescence emission spectrum of different DNA excited at different wavelength; Figure S3: Fluorescence of glucose and quinine aptamer upon titration of cortisol; Figure S4: The fluorescence emission spectrum of SYBR Green I after adding T30 and Hg^2+^; Figure S5: UV-vis spectra of adenosine; Figure S6: The fluorescence emission spectrum of adenosine; Figure S7: The fluorescence emission spectrum of the adenosine aptamer upon titration of adenosine; Figure S8: The fluorescence emission spectrum of ThT after adding 3bp and 2bp adenosine aptamer and different concentration of adenosine; Figure S9: The fluorescence emission spectrum of caffeine; Figure S10: The fluorescence emission spectra of the caffeine aptamer upon titration of caffeine; Figure S11: Fluorescence of the 1bp, 2bp and 4bp aptamer upon titration of caffeine.

## Sample Availability

Samples of the compounds are available from the authors.
