# Supplementary material for: Using the Intrinsic Fluorescence of DNA to Characterize Aptamer Binding
Source: Molecules. 2022 Nov 12;27(22):7809. doi: 10.3390/molecules27227809 (PMC9692703; doi:10.3390/molecules27227809)
Supplement: Supplementary file 1 [file molecules-27-07809-s001.zip › molecules-2005260-supplementary.pdf]

Supporting Information

Using the Intrinsic Fluorescence of DNA to Characterize Aptamer Binding

Chang Lu[1, 2], Anand Lopez[2], Jinkai Zheng[1] and Juewen Liu[2,*]

1 Institute of Food Science and Technology, Chinese Academy of Agricultural Sciences, Beijing 100193, China

2 Department of Chemistry, Waterloo Institute for Nanotechnology, University of Waterloo, Waterloo, ON, N2L 3G1, Canada

* Correspondence: liujw@uwaterloo.ca

Table S1. The DNA Sequences Used in This Work

Names	Sequences and modifications (5′–3′)
A30	AAA AAA AAA AAA AAA AAA AAA AAA AAA AAA
T30	TTT TTT TTT TTT TTT TTT TTT TTT TTT TTT
C30	CCC CCC CCC CCC CCC CCC CCC CCC CCC CCC
Cortisol aptamer (CSS.1)	GACGACGCCCGCATGTTCCATGGATAGTCTTGACTAGTCGTC
Cortisol aptamer 4bp (CSS.1-4bp)	CGACGCCAGAAGTTTACGAGGATATGGTAACATAGTCG
Cortisol aptamer 3bp (CSS.1-3bp)	GACGCCAGAAGTTTACGAGGATATGGTAACATAGTC
Adenosine aptamer (Apt_{ade})	ACC TGG GGG AGT ATT GCG GAG GAA GGT
Adenosine aptamer 3bp (Apt_{ade}-3bp)	CCT GGG GGA GTA TTG CGG AGG AAG G
Adenosine aptamer 2bp (Apt_{ade}-2bp)	CTG GGG GAG TAT TGC GGA GGA AG
Caffeine aptamer (Apt_{caff})	GAC GAC TAC GGA GTT TTA GCC GTC ACG TTC CCA GGA GTC GTC
Caffeine aptamer 1bp (Apt_{caff}-1bp)	TAC GGA GTT TTA GCC GTC ACG TTC CCA GGA
Caffeine aptamer 2bp (Apt_{caff}-2bp)	CTA CGG AGT TTT AGC CGT CAC GTT CCC AGG AG
Caffeine aptamer 3bp (Apt_{caff}-3bp)	ACT ACG GAG TTT TAG CCG TCA CGT TCC CAG GAG T
Caffeine aptamer 1bp (Apt_{caff}-4bp)	GAC TAC GGA GTT TTA GCC GTC ACG TTC CCA GGA GTC
24-mer DNA	ACGCATCTGTGAAGAGAACCTGGG
c-24-mer DNA	CCCAGGTTCTCTTCACAGATGCGT
Glucose aptamer ($Apt_{glucose}$)	ACG ACC GT TGT GTT GCT CTG TAA CAG TGT CCA TTG TCG T
Quinine aptamer (MN4)	GGC GAC AAG GAA AAT CCT TCA ACG AAG TGG GTC GCC

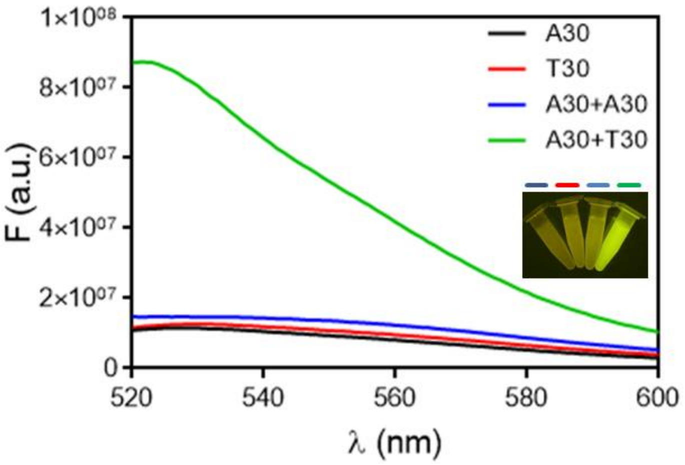

Figure S1. The fluorescence emission spectra of 100 nM ssDNA or dsDNA stained by 0.5x SGI in buffer (10 mM PB, 100 mM NaCl, pH=7), Ex=485 nm, Em=520 nm-600 nm. Inset: a photograph of the samples.

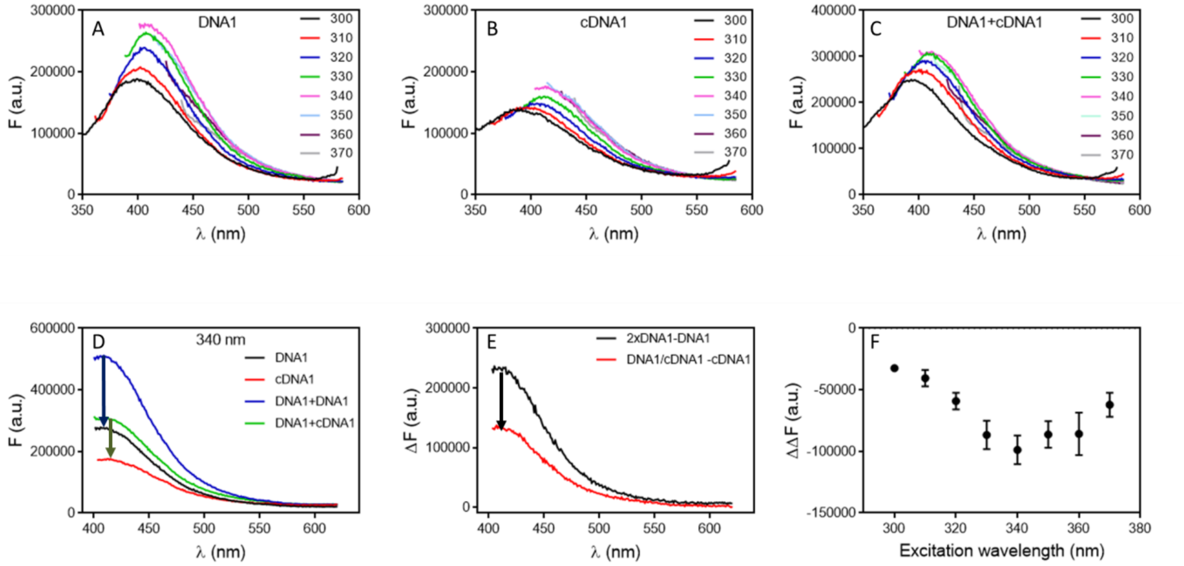

Figure S2. The fluorescence emission spectrum of 5 μM (A) 24-mer random sequenced DNA (DNA1), (B) its complementary DNA (cDNA1), and (C) double stranded DNA (DNA1+cDNA1) excited at different wavelength in buffer (10 mM PB, 100 mM NaCl, pH=7). (D) Comparison of fluorescence emission spectrum of ssDNA and dsDNA with excitation wavelength at 340 nm. (E) The fluorescence emission spectrum difference of ssDNA (2xDNA1-DNA1) and dsDNA (DNA1/cDNA1 –cDNA1) with excitation wavelength at 340 nm. (F) The maximum fluorescence difference of ssDNA and dsDNA with different excitation wavelength.

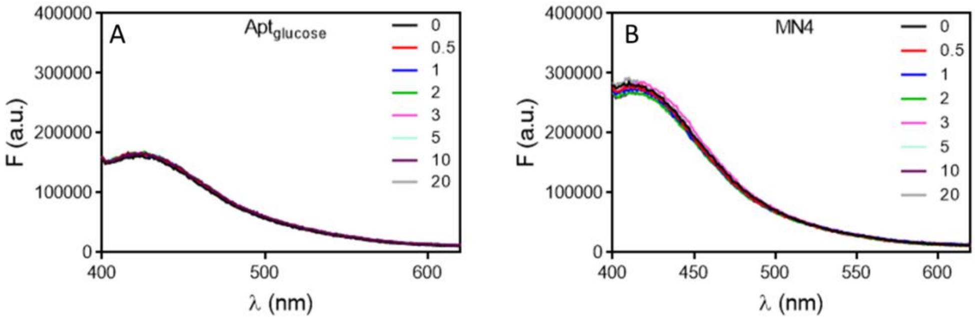

Figure S3. Fluorescence of 5 μM (A) glucose and (B) quinine aptamer upon titration of cortisol in buffer (10 mM PB, 100 mM NaCl, 10 mM $MgCl_2$ pH=7). Ex=340 nm, Em = 400 nm-620 nm.

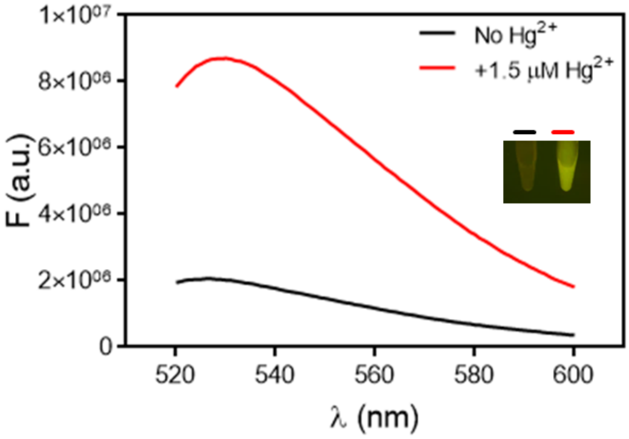

Figure S4. The fluorescence emission spectrum of 0.5×SYBR Green I after adding 100 nM T30 and 0 or 1.5 μM Hg^{2+} in buffer (10 mM PB, 100 mM $NaNO_3$, pH=7), Ex=485 nm, Em=520 nm-600 nm.

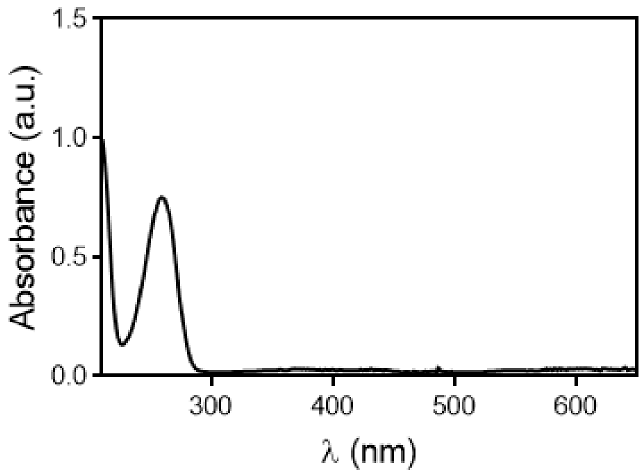

Figure S5. UV-vis spectra of 50 μM adenosine.

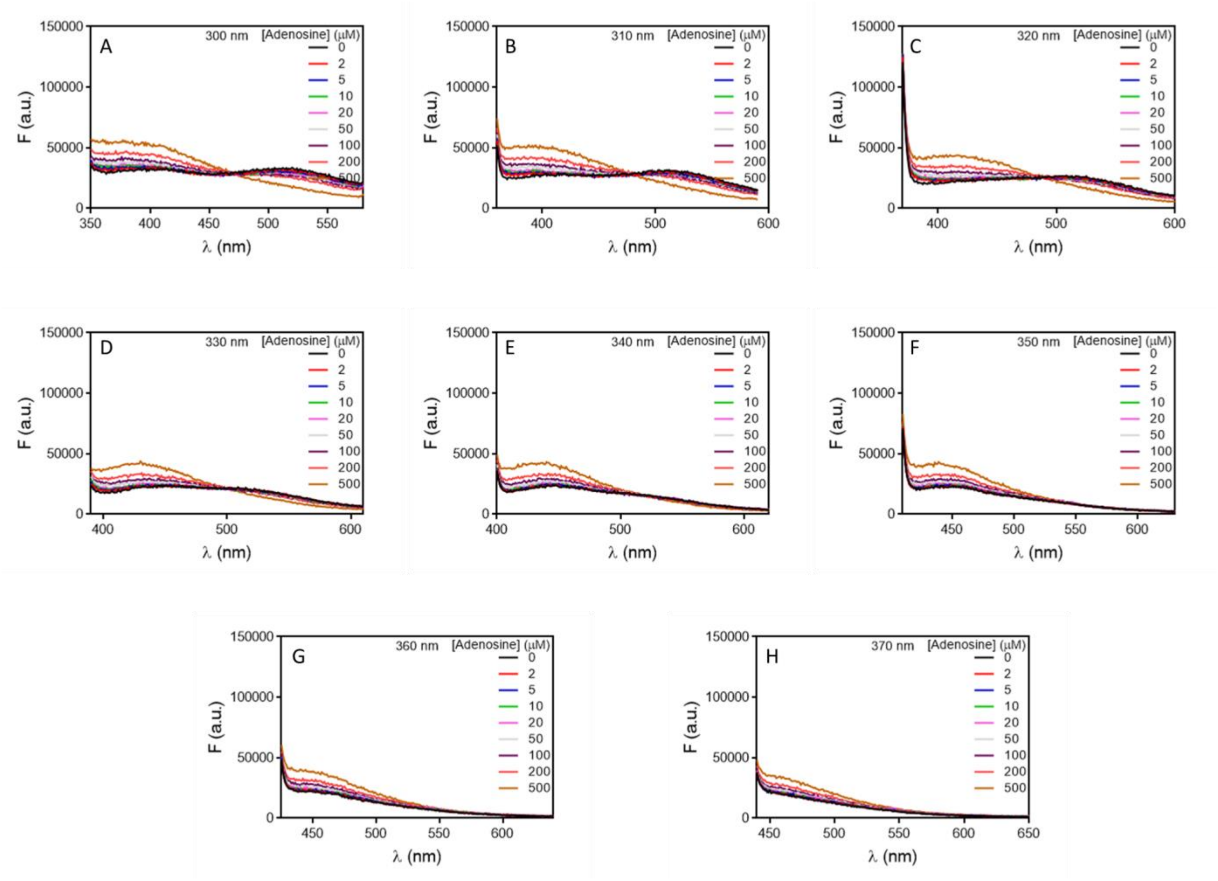

Figure S6. The fluorescence emission spectra of different concentrations of adenosine in buffer (10 mM PB, 100 mM NaCl, 10 mM $MgCl_2$ pH=7) excited by different wavelength (300 nm–370 nm).

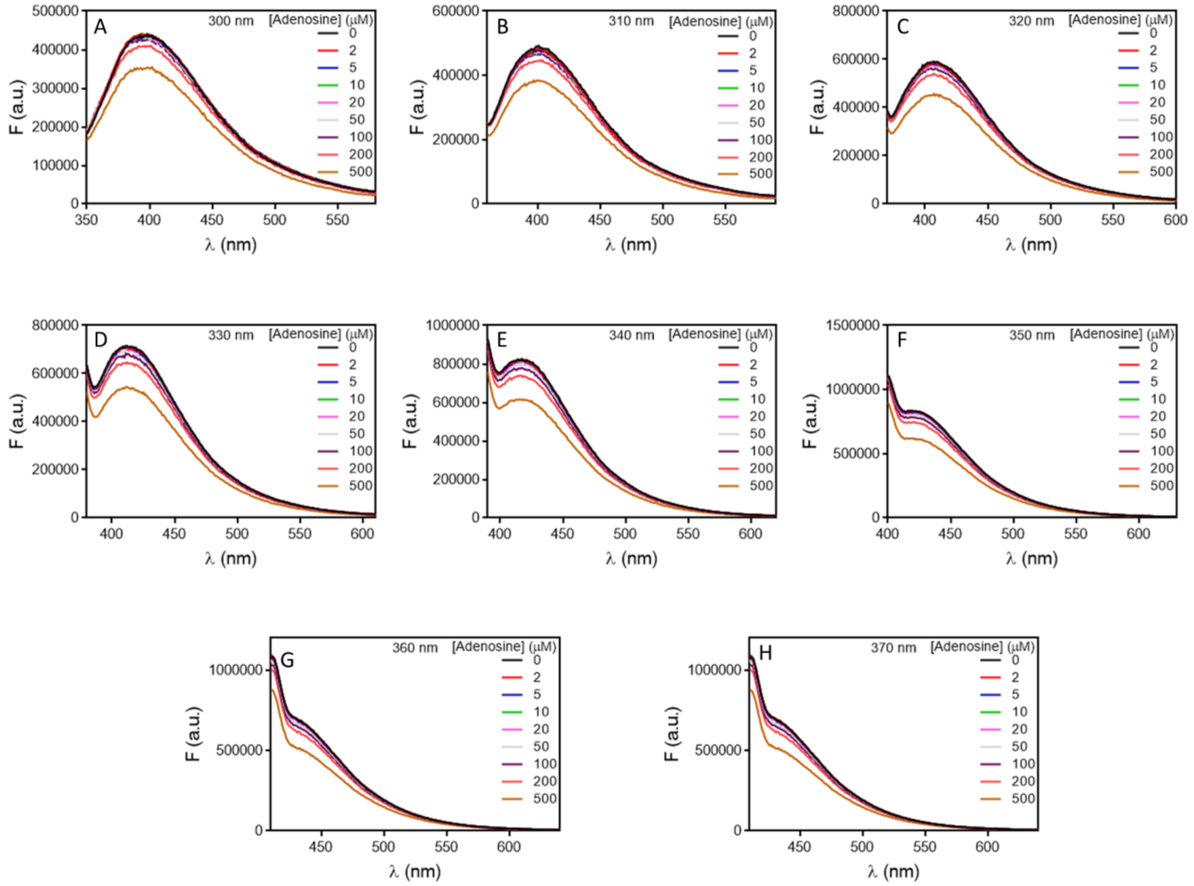

Figure S7. The fluorescence emission spectra of 5 μM of the adenosine aptamer upon titration of adenosine excited at different wavelengths (300 nm–370 nm).

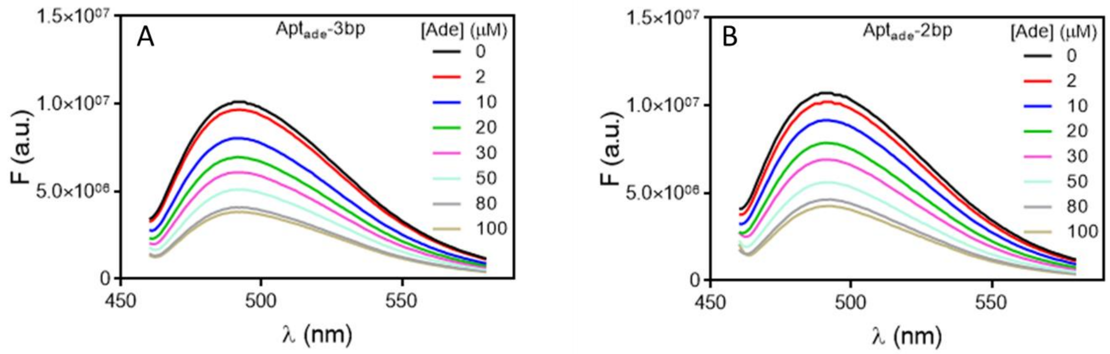

Figure S8. The fluorescence emission spectrum of 2 μM ThT after adding 100 nM (A) 3bp and (B) 2bp adenosine aptamer and different concentration of adenosine in buffer (10 mM PB, 100 mM NaCl, 10 mM $MgCl_2$ pH=7), Ex=450 nm, Em=460 nm–580 nm.

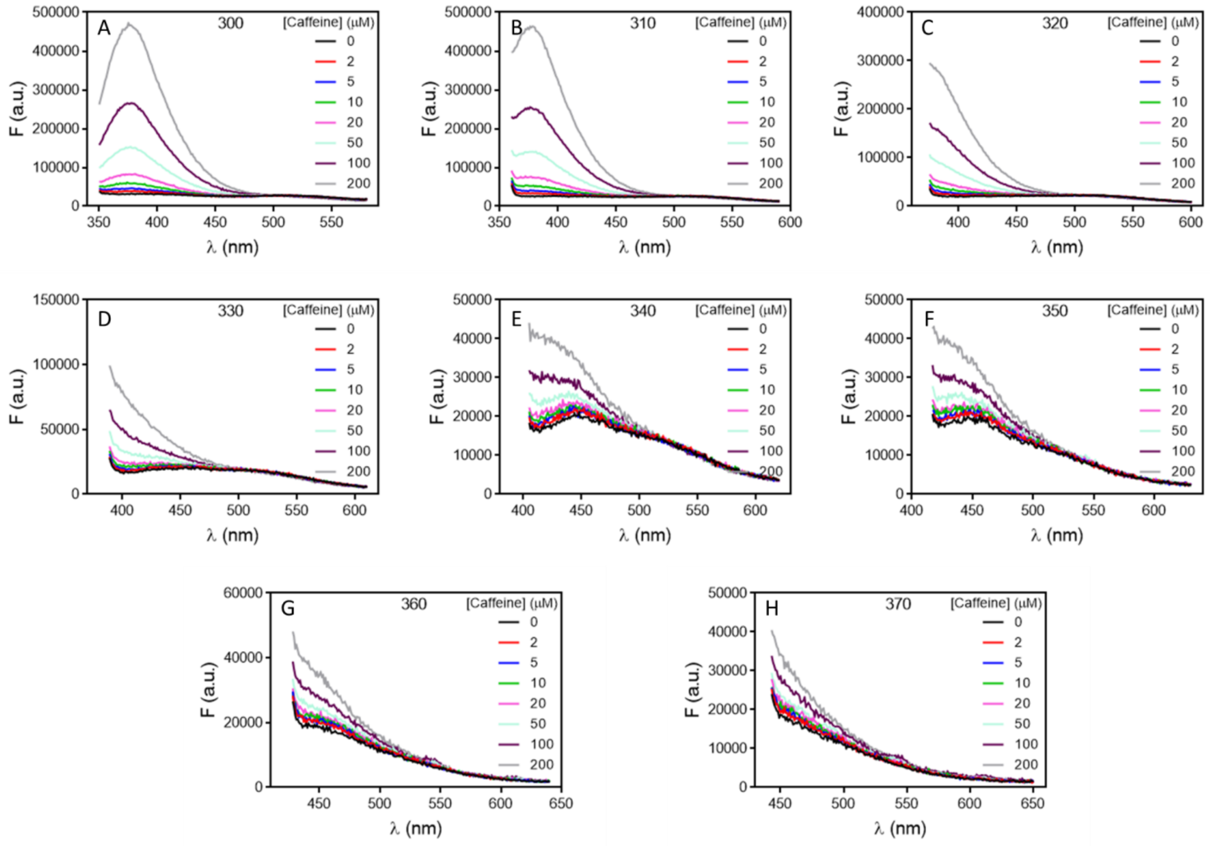

Figure S9. The fluorescence emission spectra of different concentration of caffeine in buffer (10 mM PB, 100 mM NaCl, 10 mM $MgCl_2$ pH=7) excited at different wavelengths (300 nm-370 nm).

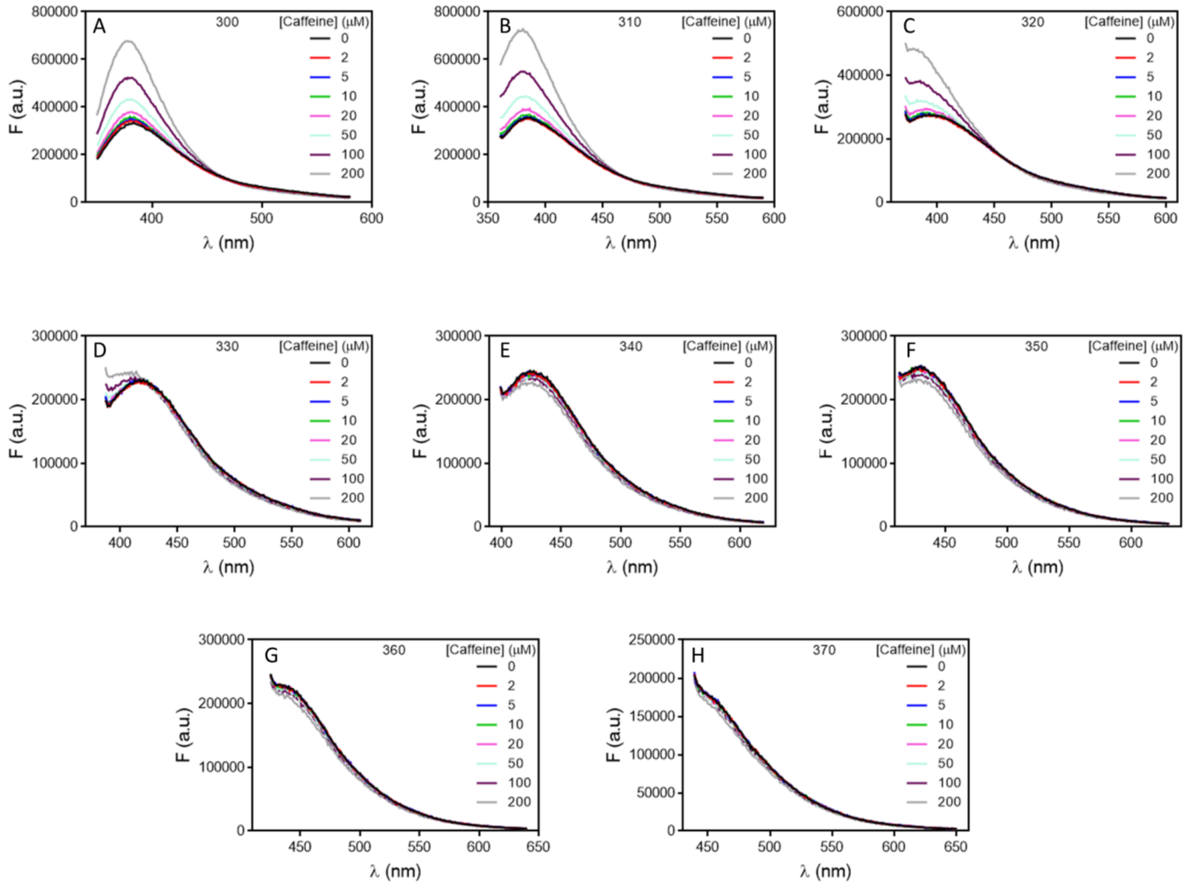

Figure S10. The fluorescence emission spectra of the caffeine aptamer (5 μM) upon titration of caffeine excited by different wavelength (300 nm-370 nm).

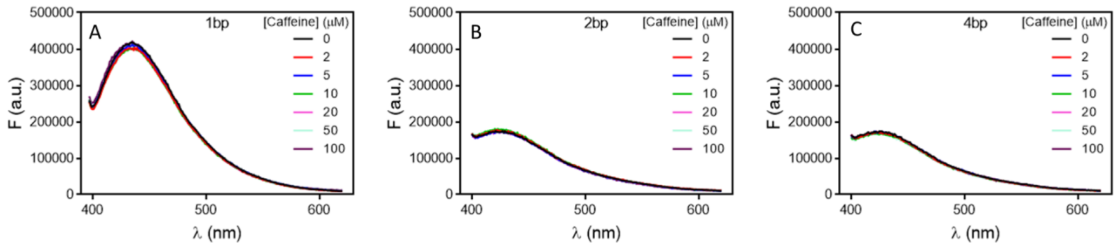

Figure S11. Fluorescence of the (A) 1bp, (B) 2bp and (C) 4bp caffeine aptamer upon titration of caffeine.
